# Comparative In Vitro Analysis of Root Cementum Surface Alterations Following Various Mechanical and Chemical Treatment Protocols in Gingival Surgery

**DOI:** 10.3390/jcm14176174

**Published:** 2025-09-01

**Authors:** Zurab Khabadze, Oleg Mordanov, Omargadzhi Magomedov

**Affiliations:** Department of Operative Dentistry, Institute of Medicine, Peoples’ Friendship University of Russia (RUDN University), Moscow 117198, Russia

**Keywords:** cementum, gingival recession, periodontics, profilometry, scanning electron microscopy (SEM), surface roughness, tissue regeneration

## Abstract

**Background/Objectives:** Gingival recession poses significant challenges in periodontal therapy, particularly in procedures aimed at achieving predictable root coverage and long-term stability of grafts. Conditioning of the root surface plays a crucial role in improving biomaterial adhesion and facilitating periodontal regeneration. This in vitro study aimed to evaluate the morphological and microroughness alterations of root cementum following different mechanical and chemical conditioning protocols commonly used in mucogingival surgery. **Methods:** Forty extracted human single-rooted teeth were randomly allocated into eight groups: untreated control, mechanical scaling alone, and scaling combined with ethylenediaminetetraacetic acid (EDTA), citric acid, phosphoric acid, tetracycline, doxycycline, or saline. Surface roughness was measured using contact profilometry, while structural modifications were analyzed via scanning electron microscopy. **Results:** Statistically significant intergroup differences (*p* < 0.05) were observed. Baneocin treatment produced the most conservative changes, with limited surface roughness and minimal structural alteration, whereas phosphoric acid, tetracycline, and EDTA caused pronounced demineralization and surface porosity. Citric acid and doxycycline induced moderate alterations, with partial preservation of cementum integrity. The null hypothesis assuming no surface or morphological changes was rejected. **Conclusions:** These findings indicate that low-aggressiveness agents may achieve an optimal balance between surface decontamination and cementum preservation, which is critical for enhancing graft integration and improving clinical outcomes in root coverage surgery.

## 1. Introduction

Gingival recession, characterized by the apical displacement of the gingival margin with concomitant root surface exposure, presents both functional and esthetic challenges. Clinically, it is associated with an increased risk of dentin hypersensitivity, root caries, and plaque accumulation. Additionally, it exerts a significant psychological and cosmetic impact on affected individuals. Successful surgical management of gingival recession—particularly through coronally advanced flaps (CAFs) combined with connective tissue grafts (CTGs)—requires meticulous root surface preparation to ensure favorable clinical outcomes [1,2,3].

Root surface treatment plays a pivotal role in achieving optimal biological attachment and long-term stability of soft tissue grafts. Conventional root planing using curettes allows for the removal of bacterial biofilm and necrotic cementum; however, it may also lead to the formation of a smear layer that hinders healing. To enhance biological adaptation, various chemical and antimicrobial agents—such as tetracycline, ethylenediaminetetraacetic acid (EDTA), citric acid, and phosphoric acid—have been proposed. These agents aim to modify the cementum’s microrelief and expose dentinal tubules, potentially improving the conditions for tissue regeneration [4,5].

Nevertheless, the effects of these agents differ significantly in terms of pH levels, chelating capacity, and tissue interactions. While highly acidic solutions such as phosphoric acid can cause excessive demineralization, milder agents may preserve cementum structure while achieving sufficient cleansing. Excessive trauma or degradation of the cementum matrix may impair graft adaptation, particularly in the presence of micro-erosive damage [4,5].

Modern techniques such as scanning electron microscopy (SEM) and contact profilometry offer precise tools for assessing the morphological alterations induced by various treatment protocols. These methods facilitate not only roughness measurement but also the evaluation of surface continuity, porosity, and architectural integrity—factors critical to soft tissue attachment and healing [6].

While several studies have assessed chemical root conditioning, direct comparative evidence across multiple agents, including antibiotics, remains limited. This study addresses this gap by simultaneously evaluating eight distinct protocols using standardized profilometric and SEM methodologies, thus providing novel insights into the balance between decontamination efficacy and cementum preservation.

The objective of this study was to perform a comparative analysis of mechanical and chemical root surface treatment protocols used in the context of surgical coverage of gingival recessions. The effects of different agents—including EDTA, citric acid, phosphoric acid, tetracycline, doxycycline, and saline—on root cementum microstructure, surface roughness, and morphological integrity were evaluated. The study aimed to identify protocols that promote favorable conditions for soft tissue adaptation while highlighting aggressive factors associated with demineralization and structural damage. Although several chemical agents have been tested, direct comparative data across multiple conditioning methods—including antibiotics—remain scarce. This lack of evidence complicates clinical decision-making. Our study provides a standardized comparative analysis to address this gap.

To guide the analysis, the following null hypotheses were formulated:

**H_1_.**  *There is no statistically significant difference in root cementum surface roughness between mechanical instrumentation alone and its combination with chemical conditioning.*

**H_2_.**  *The use of different chemical agents does not result in significant structural alterations (e.g., erosion, demineralization, or porosity) of the root surface compared to mechanical treatment alone.*

## 2. Materials and Methods

### 2.1. Sample Selection and Group Allocation

This in vitro study was conducted using extracted human third molars collected for orthodontic indications. Written informed consent for the use of biological material was obtained from all donors prior to extraction. This study was conducted in accordance with the institutional policy of RUDN University (Ethic Protocol No. 18987, 18 May 2025) and international guidelines, including the World Medical Association’s Declaration of Helsinki and the ICH-GCP recommendations.

A total of 40 extracted single-rooted human teeth with intact root surfaces were selected for this in vitro study. Only single-rooted third molars extracted for orthodontic indications were included. All specimens were stored in a 0.1% thymol solution at 4 °C and utilized within three months post-extraction. Inclusion criteria included the absence of carious lesions, restorations, root fractures, or any signs of prior periodontal treatment.

**Figure 1:**
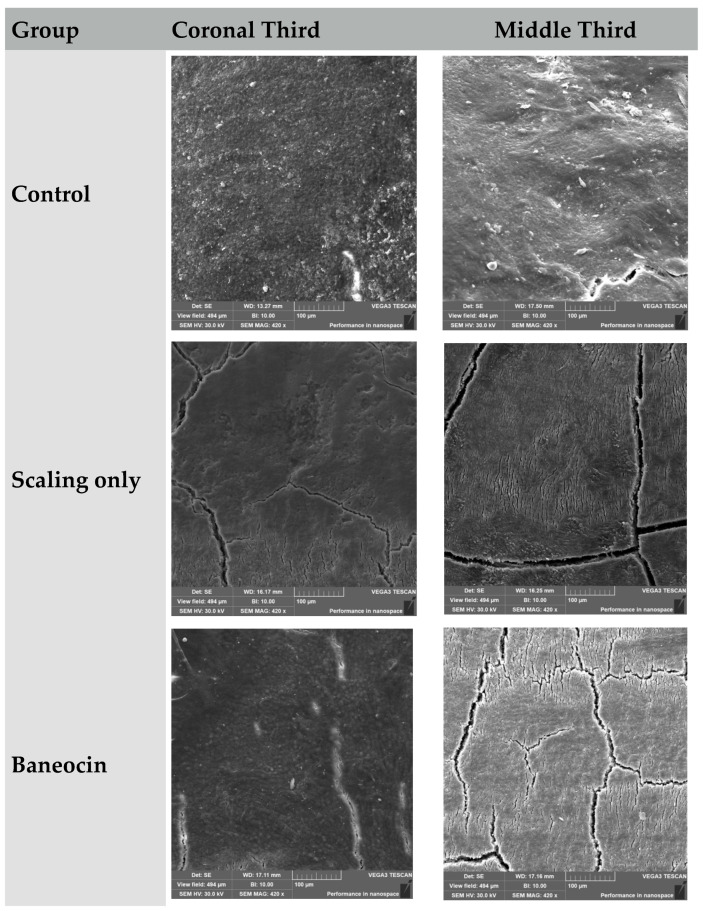

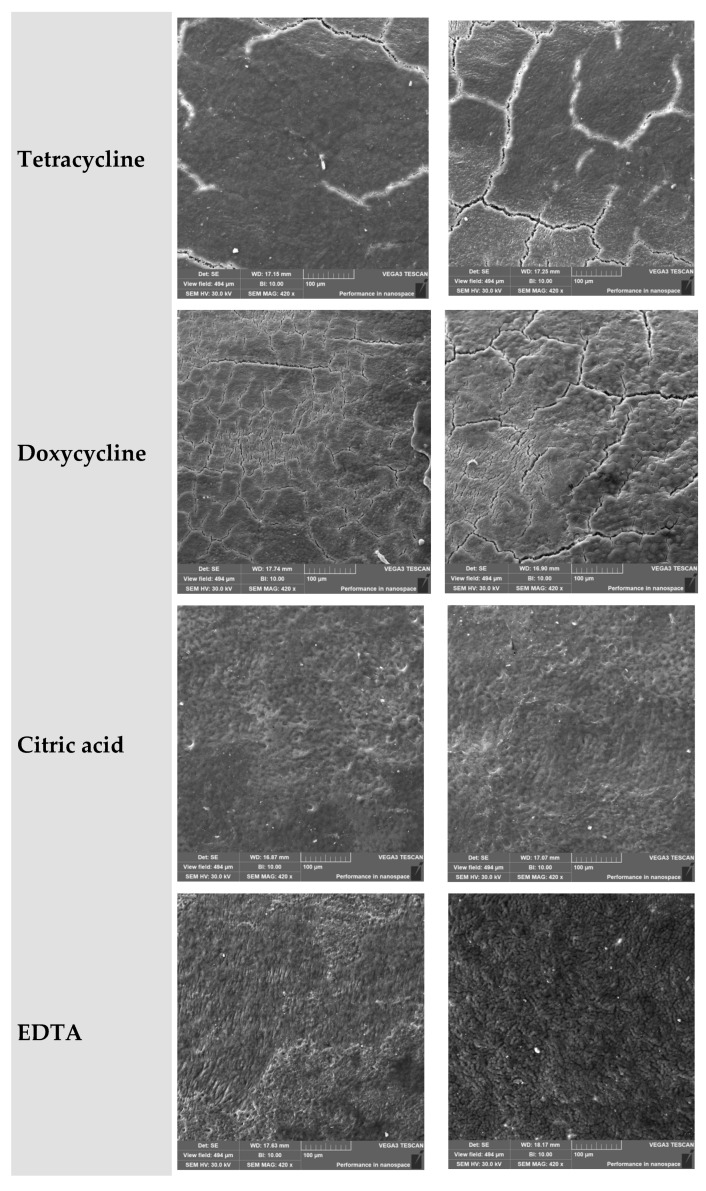

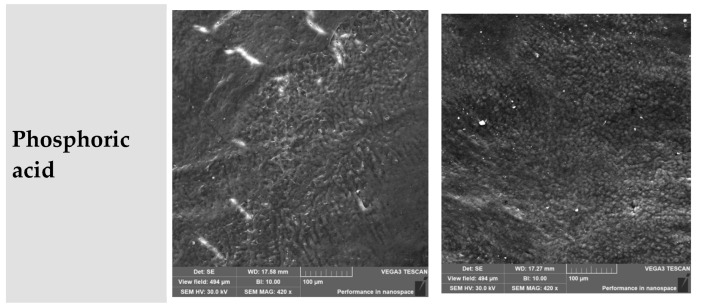
Comparative SEM Evaluation of Root Surface Morphology in the Coronal and Middle Thirds Following Various Treatment Protocols.

**Figure 2:**
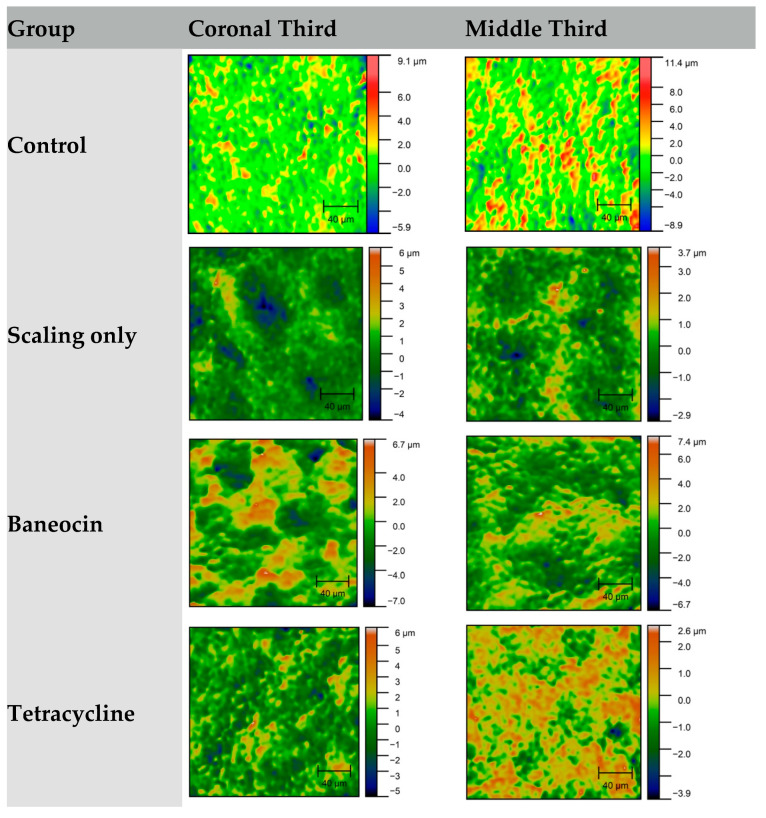

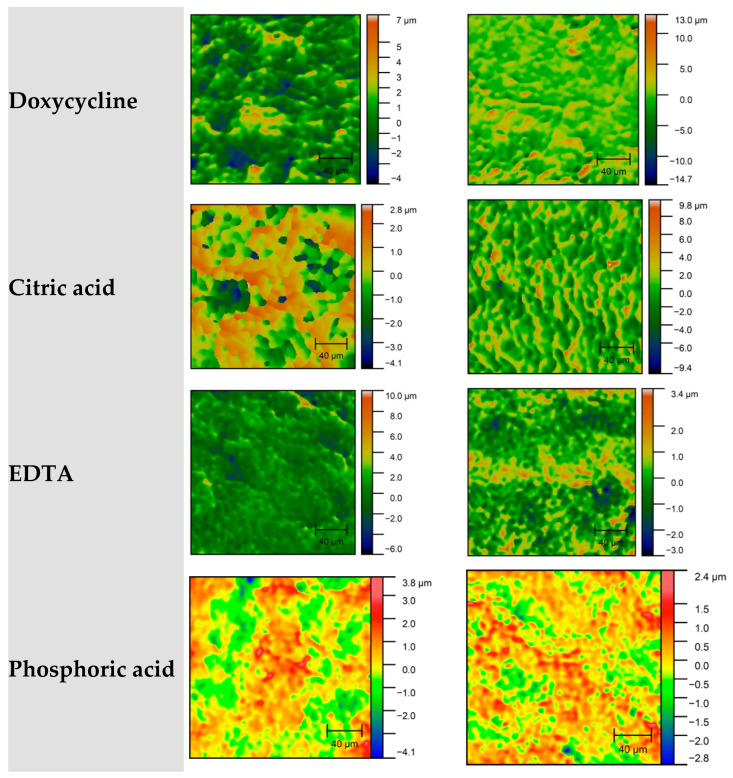
Comparative Prophylometry Evaluation of Root Surface Morphology in the Coronal and Middle Thirds Following Various Treatment Protocols.

**Figure 3:**
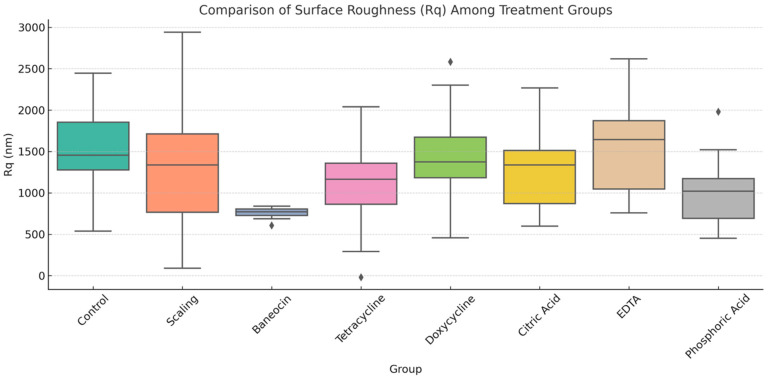
Boxplot Comparison of Root Surface Roughness (Rq) Among Experimental Groups. ♦ Rhombus indicates outlier values that deviate significantly from the main data distribution.

**Figure 4:**
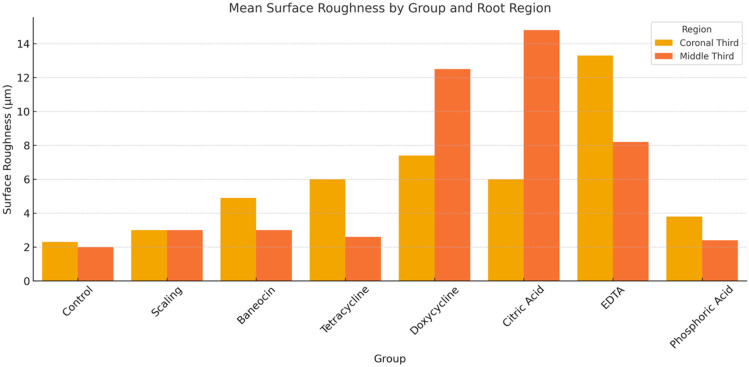
Mean Surface Roughness by Group and Root Region.

**Figure 5:**
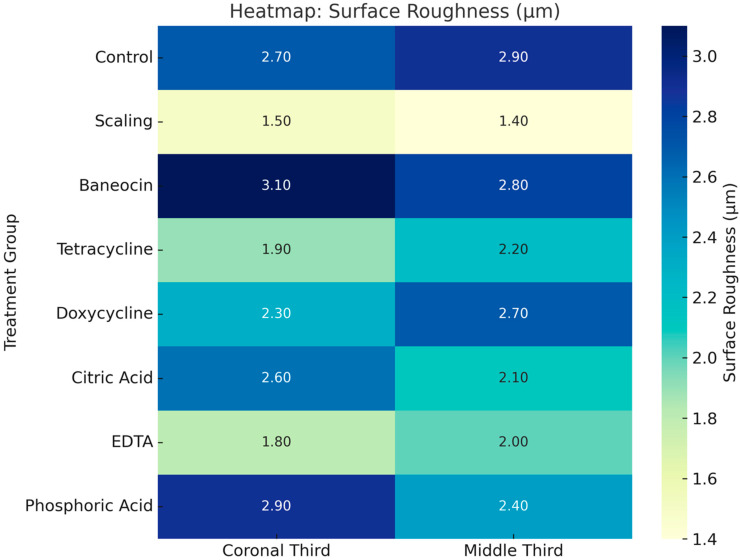
Heatmap of Surface Roughness (µm) by Group and Root Region.

The samples were randomly assigned to eight experimental groups (n = 5 per group) based on the type of mechanical and/or chemical surface treatment applied to the root (see Figure 1, Figure 2, Figure 3, Figure 4 and Figure 5):

- Group 1 (Intact control): No treatment was applied to the root surface. These samples served as a baseline reference for morphological and topographical comparison.
- Group 2 (Scaling only): Manual debridement was performed using Gracey curettes (Hu-Friedy, Chicago, IL, USA) under light pressure (~200 g), with vertical and horizontal strokes for 30 s.
- Group 3 (Scaling + EDTA): After mechanical instrumentation, 24% EDTA gel (MD-ChelCream, Meta Biomed, Colmar, PA, USA) was applied to the root surface for 2 min and rinsed with sterile distilled water.
- Group 4 (Scaling + citric acid): A 15% citric acid solution (pH ≈ 1.0) was applied using a microbrush for 2 min following scaling, then thoroughly rinsed.
- Group 5 (Scaling + phosphoric acid): A 37% phosphoric acid gel (Dental Etch, 3M ESPE, Seefeld, Germany) was applied for 15 s post-scaling and rinsed with sterile saline.
- Group 6 (Scaling + tetracycline): A 10% tetracycline hydrochloride solution was applied using a cotton pellet for 3 min and then rinsed.
- Group 7 (Scaling + doxycycline): A 10% doxycycline solution was used following the same protocol as in Group 6.
- Group 8 (Scaling + saline): After mechanical treatment, a 0.9% NaCl solution was applied to the root surface for 2 min to assess potential neutral effects.

All treatments were standardized and performed on the coronal and middle thirds of the root. Following treatment, each sample was stored in a sealed, humidified chamber (100% relative humidity) for 24 h prior to analysis.

### 2.2. Surface Roughness Measurement

Quantitative evaluation of surface roughness was conducted using a contact profilometer (Surftest SJ-210, Mitutoyo, Kawasaki, Japan). Measurements were taken at three distinct points on each sample, with the stylus moving perpendicularly to the direction of instrumentation. The following parameters were recorded:

- Ra—arithmetic mean deviation of the profile;
- Rz—average peak-to-valley height;
- Rq—root mean square roughness.

For each parameter, group means were calculated and used for intergroup comparison.

### 2.3. Scanning Electron Microscopy (SEM)

Specimens were fixed in 2.5% glutaraldehyde solution, dehydrated in a graded ethanol series, air-dried, and sputter-coated with gold. Microstructural analysis was performed using a Phenom XL scanning electron microscope (Thermo Fisher Scientific, Waltham, MA, USA) at 420× magnification. Evaluation criteria included the presence or absence of a smear layer, degree of surface porosity, evidence of erosive damage, and overall cementum structural organization.

### 2.4. Statistical Analysis

Data distribution normality was assessed using the Shapiro–Wilk test. One-way analysis of variance (ANOVA) was employed to determine intergroup differences in roughness parameters (Ra, Rz, Rq), followed by Bonferroni post hoc correction. Statistical significance was set at *p* < 0.05. All analyses were performed using SPSS software version 25.0 (IBM Corp., Armonk, NY, USA).

## 3. Results

### 3.1. Scanning Electron Microscopy (Figure 1)

The scanning electron microscopy (SEM) analysis of root surfaces revealed distinct differences in microstructure, surface roughness, and the extent of tissue alteration following the application of various treatment protocols.

### 3.2. Control Group (Untreated)

The root surface remained smooth, with minimal microroughness characteristic of intact cementum. Dentin tubules were entirely closed, and the overall structure appeared continuous and undamaged. This group served as the baseline for morphological comparison.

- Coronal third: Preserved morphology with moderate physiological roughness.
- Middle third: Slightly increased granularity without notable structural damage.

### 3.3. Scaling Only

Mechanical instrumentation resulted in pronounced removal of cementum, exposure of dentin, and formation of surface grooves. The surface became markedly rougher, exhibiting multiple cracks and signs of disrupted architecture. Although dentinal tubules were exposed, areas of potential hypersensitivity were also evident.

- Coronal third: Evidence of trauma, linear defects, and partial cementum removal.
- Middle third: Loosely organized surface, structural breakdown of the cementum layer.

### 3.4. Baneocin

This antibiotic exhibited a moderate effect on the root surface. Partial demineralization and a slight increase in surface roughness were observed. Despite some microstructural disruption, zones of intact cementum were preserved, suggesting that Baneocin may serve as a gentle antimicrobial treatment option.

- Coronal third: Granular surface with moderate structural alteration.
- Middle third: Deeper zones of destruction and porosity.

### 3.5. Tetracycline

Treatment with tetracycline resulted in noticeable surface roughening and localized porosity. Moderate etching and tubule exposure were noted. Due to its antibacterial properties, tetracycline may be appropriate for decontamination protocols.

- Coronal third: Reticulated microtexture with initial signs of degradation.
- Middle third: Extensive etching and cementum destruction.

### 3.6. Doxycycline

Compared to tetracycline, doxycycline had a more aggressive impact. SEM images showed distinct structural damage, irregular surface texture, and marked exposure of dentinal tubules. Cementum breakdown and uneven morphology were prominent, highlighting the need for cautious use to avoid hypersensitivity.

- Coronal third: Partial structural disruption with remnants of original morphology.
- Middle third: Increased granularity and loss of uniform surface characteristics.

### 3.7. Citric Acid

Citric acid caused pronounced etching of the root surface. High microroughness, deep erosions, and a disorganized surface with collagen exposure were evident. These features make citric acid effective for preparing the root for regeneration.

- Coronal third: Fine-grained and well-organized surface morphology.
- Middle third: Porosity present, but the general structure remained preserved.

### 3.8. EDTA

Application of EDTA moderately disrupted the smear layer and exposed dentinal tubules. The microstructure remained relatively organized, suggesting its suitability for controlled chemical conditioning prior to adhesion or remineralization.

- Coronal third: Surface varied from mild etching to a reticulated pattern.
- Middle third: Loosely arranged and extensively demineralized area.

### 3.9. Phosphoric Acid (H_3_PO_4_)

Phosphoric acid proved to be the most aggressive agent. The surface was heavily damaged, with full exposure of dentinal tubules and areas of cementum fragmentation. While potentially effective for deep cleansing, this method carries a high risk of excessive tissue loss.

- Coronal third: Ranged from partial to deep etching, depending on the subgroup.
- Middle third: Severely degraded in all specimens.

Overall, Table 1 and Table 2 illustrate the hierarchical impact of treatments on root surface morphology, ranging from physiologically compatible modifications to overtly destructive outcomes. These findings highlight the critical importance of balancing surface conditioning efficacy with preservation of cementum integrity in root coverage procedures.

Based on the statistically significant result of the Kruskal–Wallis test, it can be concluded that surface roughness varied substantially among the treatment groups. The group treated with Baneocin exhibited the lowest roughness values and the most uniform surface morphology (mean ~750 nm), indicating a stable and controlled effect. In contrast, tetracycline and EDTA demonstrated the highest surface roughness values and the greatest variability, suggesting a potentially erosive and unpredictable influence on the root surface. Treatments with phosphoric acid, citric acid, and doxycycline produced intermediate roughness values and were characterized by a balanced effect on surface modification (Figure 3).

Statistical analysis confirmed significant intergroup differences in root surface roughness (*p* = 0.001, Kruskal–Wallis test). The Baneocin group demonstrated the lowest Rq values with minimal variation, supporting its gentle and consistent action. In contrast, groups treated with tetracycline and EDTA exhibited the highest Rq values with a broad standard deviation, pointing to their aggressive and less controlled impact on the cementum. More moderate and structurally preserving effects were observed in the citric acid, phosphoric acid, and doxycycline groups, which increased surface roughness to a controlled degree without damaging the original surface morphology (Table 3). These findings highlight the importance of selecting biomodification agents not only based on their ability to increase surface microroughness but also in relation to their preservation of morphological integrity.

Profilometric analysis demonstrated that variations in micro-roughness between the coronal and middle thirds of the root surface were group-dependent (Figure 4 and Figure 5). In the control group, roughness values were moderately consistent between both regions. In contrast, scaling resulted in reduced roughness in the middle third, possibly due to the limited depth of instrument action. The group treated with Baneocin exhibited higher roughness values in the coronal third, which may be attributed to residual material or its specific penetration characteristics. Doxycycline demonstrated greater roughness values and more extensive cementum disruption compared to tetracycline, suggesting a stronger demineralizing effect.

Citric acid and EDTA had a more pronounced effect on the coronal third, increasing micro-roughness in that region, presumably as a result of acidic dissolution of the superficial cementum layer. In the phosphoric acid group, differences between the two root thirds were less marked; however, the overall roughness values remained high, reflecting the agent’s aggressive etching capacity. In most groups, the middle third exhibited either reduced or stabilized roughness levels compared to the coronal third, suggesting a limited depth of chemical penetration or differences in cementum morphology.

Collectively, these findings emphasize the importance of evaluating the localized effects of chemical agents at different root levels to optimize clinical outcomes while minimizing the risk of unnecessary tissue damage.

## 4. Discussion

The current in vitro study provided a comprehensive comparison of root cementum alterations following various mechanical and chemical surface treatments, highlighting significant intergroup differences in micromorphology and roughness profiles. Mechanical scaling alone caused notable physical trauma, including microfractures and structural disintegration, supporting prior concerns regarding the aggressiveness of conventional root planing [1]. SEM and profilometric analyses revealed that adjunctive chemical treatments influenced surface texture and architecture in highly agent-specific patterns. These findings support the notion that root biomodification protocols should balance cleansing efficacy with preservation of the cementum substrate [2,3,7]. The observed surface variability across regions further reinforces the importance of regional root morphology in clinical decision-making.

Among the chemical agents studied, Baneocin exhibited the most conservative impact, with consistently low roughness values and minimal morphological disruption. This suggests its potential as a gentle antimicrobial adjunct that preserves the cementum’s integrity—an essential factor for promoting stable connective tissue attachment [3]. In contrast, agents such as tetracycline and EDTA resulted in high surface roughness values and variable microdamage, indicating uncontrolled erosion and unpredictable outcomes [2,6]. These findings challenge the routine use of aggressive chelators for root conditioning without careful consideration of their depth of penetration and destructive potential. The data support a tailored application depending on the clinical context, rather than uniform adoption of standard protocols.

The retrospective analysis by Górski and Szerszeń [8] examined the adjunctive use of 24% EDTA and enamel matrix derivative (EMD) in combination with the modified coronally advanced tunnel (MCAT) technique and subepithelial connective tissue grafts (SCTG) for the treatment of multiple gingival recessions. Despite significant within-group improvements across all clinical parameters at 12 months, intergroup differences in average and complete root coverage (ARC and CRC) did not reach statistical significance (*p* > 0.05). Notably, only clinical attachment level (CAL) gain and esthetic parameters based on the Root Coverage Esthetic Score (RES) showed statistically superior outcomes in the EDTA + EMD group compared to EDTA or saline controls. These findings underscore the limited additive benefit of root surface biomodification on root coverage extent per se, while suggesting potential enhancement in soft tissue integration and CAL stability following the adjunctive application of EMD.

Baher et al. [9] conducted a well-controlled in vivo investigation into the histological effects of EDTA application following scaling and root planing (SRP) in a canine model. Their findings demonstrated that root biomodification with 24% EDTA significantly enhanced the early proliferation of fibroblasts and collagen fiber insertion within the connective tissue attachment zone, as opposed to SRP alone. However, no additional improvement in clinical attachment level or junctional epithelium positioning was noted after 8 weeks, indicating that EDTA’s impact may be predominantly limited to early healing phases. These results provide important mechanistic insight into the role of chelating agents, supporting their utility in promoting biologically favorable root surfaces, while also emphasizing the necessity of correlating histological improvements with long-term clinical outcomes.

Citric acid and doxycycline demonstrated intermediate outcomes, with controlled etching and moderate increases in surface microroughness. Citric acid, in particular, produced a fibrillar surface ideal for connective tissue attachment while preserving the structural framework of the cementum [7,9,10]. This suggests its utility in regenerative procedures where biocompatibility and matrix exposure are prioritized. Doxycycline’s greater variability, however, raises concerns regarding dosing and contact time, which may alter its effects from regenerative to destructive [11]. These results underline the necessity of optimizing application protocols to harness benefits while minimizing unintended side effects.

The randomized controlled study by Maruyama et al. [12] compared the efficacy of Er:YAG laser and EDTA application on root surfaces in the context of coronally advanced flap (CAF) procedures. Although both modalities resulted in statistically significant improvement in clinical attachment level (CAL), keratinized tissue width, and root coverage after six months, no significant intergroup differences were observed. Notably, SEM evaluation revealed that Er:YAG laser irradiation produced a clean, micro-irregular surface free of smear layer and carbonization, while EDTA left residual organic debris. These findings suggest that laser-assisted root conditioning may offer a predictable alternative to chemical agents, ensuring effective decontamination and topographic modification without compromising cementum integrity—an aspect particularly relevant when aiming to avoid over-etching or cytotoxic effects associated with low-pH agents.

Phosphoric acid, while effective in smear layer removal, induced severe damage, including cementum loss and dentinal tubule exposure in both coronal and middle thirds [13]. This excessive demineralization poses a risk of hypersensitivity and impaired healing, which may undermine the goals of root coverage procedures. The findings corroborate previous literature describing the destructive potential of strong acids on dentin and cementum, particularly when applied for prolonged periods or at high concentrations [14]. Clinicians should exercise caution in using such agents, especially when structural preservation is critical to clinical success. Given its aggressive action, phosphoric acid may be more appropriate in restorative rather than periodontal regenerative contexts.

Evaluation of the null hypotheses further supports the interpretation of the results. The first null hypothesis (H_1_), which posited no difference in surface roughness between mechanical and chemical protocols, was rejected based on statistically significant differences (*p* < 0.05) in profilometric outcomes. The second null hypothesis (H_2_), assuming no structural alterations following chemical treatment, was likewise rejected in light of SEM evidence showing erosion, porosity, and surface breakdown in several groups [2,6]. These rejections validate the hypothesis that chemical conditioning produces measurable and heterogeneous impacts on cementum morphology. The outcomes confirm that treatment selection must be guided by both empirical evidence and clinical objectives.

Despite the controlled experimental environment, the study has several limitations. First, as an in vitro investigation, it lacks the biological variables and healing dynamics present in a living periodontium, including saliva, gingival crevicular fluid, and patient-specific immune responses [15]. Second, the standardized treatment durations may not fully reflect the variability of chairside application in clinical practice. Third, while SEM and profilometry provide detailed surface characterization, they do not directly assess biological responses such as fibroblast adhesion or collagen fiber orientation [4]. Further in vivo and histological studies are needed to confirm the clinical relevance of the observed surface alterations.

Finally, while this study focused on physical and morphological outcomes, future investigations should integrate biological endpoints, such as fibroblast proliferation, gene expression, or wound healing dynamics [15]. Combining morphological analyses with cell-based assays could provide a more holistic view of the impact of surface treatments on periodontal regeneration. Moreover, long-term studies evaluating clinical parameters such as root coverage success, patient comfort, and recurrence rates would enhance translational relevance [2]. Interdisciplinary approaches incorporating molecular biology, materials science, and clinical periodontology are warranted to refine surface conditioning strategies. Such integration may pave the way for evidence-based guidelines that maximize therapeutic success in root coverage surgeries. A further limitation of this study is the relatively small sample size (five teeth per group), which reduces statistical power and generalizability. Future in vivo studies with larger sample cohorts are essential to corroborate these observations.

## 5. Conclusions

This in vitro comparative study demonstrated that different mechanical and chemical root surface conditioning protocols exerted highly variable effects on cementum morphology and microroughness. Among the tested agents, phosphoric acid, EDTA, and tetracycline produced the most aggressive alterations, characterized by marked demineralization, porosity, and structural breakdown. Citric acid and doxycycline induced moderate etching with partial preservation of cementum integrity, while Baneocin and saline proved to be the most conservative treatments, maintaining structural continuity with minimal increases in surface roughness.

From a clinical perspective, these findings emphasize the importance of balancing effective smear layer removal and surface modification with the preservation of cementum integrity to support favorable soft tissue healing in root coverage procedures. Agents that cause excessive demineralization, such as phosphoric acid, should be used with caution, whereas milder options may provide safer alternatives.

The study’s limitations include the small sample size (n = 5 per group), the in vitro design, and the absence of biological outcome measures such as fibroblast adhesion or histological healing. These constraints limit the generalizability of the results. Future research should therefore focus on larger-scale in vivo and histological studies to validate these observations and translate them into clinical recommendations.

## Figures and Tables

**Table 1:** Summary of Root Surface Characteristics Following Various Treatment Protocols.

Group	Surface Condition	Roughness	Microstructure	Conclusion
Control	Intact, smooth	Minimal	Continuous, unchanged	Reference surface for comparison
Scaling	Rough, grooves, exposed dentin	High	Cracks, microfractures	Mechanical damage
Baneocin	Moderately altered, partially cleaned	Medium	Mixed structure, preserved cement zones	Moderate structural alteration with partial preservation
Tetracycline	Rough, partially etched	Moderate	Porous, exposed tubules	Balanced effect
Doxycycline	Deeply damaged, loose	High	Severe cementum degradation	Caution required
Citric acid	Etched, erosive, collagen exposed	Very high	Porosity, fibrillar texture	Optimal for regeneration
EDTA	Smear layer removed, moderate etching	Significant	Organized tubules, preserved architecture	Controlled treatment
Phosphoric acid	Destruction, full tubule exposure, deep erosions	Excessive	Matrix breakdown, fiber separation, loss of continuity	Aggressive agent, high damage risk

**Table 2:** Comparative Summary of Root Surface Characteristics in Coronal and Middle Thirds Following Various Treatment Protocols.

Group	Coronal Third	Middle Third	Comparative Conclusion
Control	Homogeneous, intact cementum structure, moderate roughness	Preserved structure, increased granularity, minor defects	Structure mostly preserved, cementum intact, physiologic surface
Scaling	Surface etching, partial cementum exposure, linear defects	Loose, fragmented, signs of leaching and cementum erosion	Traumatic intervention causes structural damage and leaching
Baneocin	Segmented structure, presence of granules and roughness	Porous, extensive destruction, granular texture	Antibiotic induces destructive changes in both thirds
Tetracycline	Fine-grained with microcracks, surface breakdown	Cementum disintegration, chaotic and porous	Pronounced destruction in middle third, aggressive leaching
Doxycycline	Dense with preserved morphology, signs of surface degradation	Granular and loose, signs of demineralization, partial destruction	Progressive degradation from coronal to middle third, moderate effect
Citric acid	Fine-grained, uniform, crack-free, moderate roughness	Porous but preserved morphology, uniform roughness	Gentle impact, structure largely preserved
EDTA	Segmented or degraded, from ‘orange peel’ texture to complete destruction	Loose to nearly completely degraded surface	Maximum degradation in middle third, cementum lost
Phosphoric acid	Ranging from relatively smooth to fully degraded; shallow to deep erosion	Loose, granular, variable depth of cementum destruction	Phosphoric acid induces aggressive demineralization, especially in depth

**Table 3:** Comparative Analysis of Surface Roughness (Rq) Values Across Treatment Groups.

Group	Mean Rq (nm)	SD Rq (nm)	Description
Group 1—Control	1583.25	546.73	No treatment
Group 2—Scaling	1556.61	748.22	Mechanical instrumentation (second control)
Group 3—Baneocin	753.4	55.84	Scaling + bacitracin
Group 4—Tetracycline	1161.78	594.03	Scaling + tetracycline
Group 5—Doxycycline	1387.44	485.4	Scaling + doxycycline
Group 6—Citric Acid	1305.94	439.6	Scaling + citric acid
Group 7—EDTA	1476.94	602.61	Scaling + EDTA
Group 8—Phosphoric Acid	998.22	360.42	Scaling + H_3_PO_4_

## Data Availability

The original contributions presented in this study are included in the article. Further inquiries can be directed to the corresponding author.
